# An Investigation Into the Risk Factors for Transfusion Need After Neck of Femur Fracture Surgery

**DOI:** 10.7759/cureus.67673

**Published:** 2024-08-24

**Authors:** Abdullah Bin Sahl, Emma Biggs, Upamanyu Nath, Abdelwakeel Bakhiet, Tom Collins, Anand Pillai

**Affiliations:** 1 Trauma and Orthopaedics, Wythenshawe Hospital, Manchester University NHS Foundation Trust, Manchester, GBR; 2 Trauma and Orthopaedics, University of Manchester, Manchester, GBR

**Keywords:** risk, transfusion, blood loss, hip, fracture, neck of femur

## Abstract

Background: A common surgical procedure in the elderly is the operation on neck of femur fractures, with a primary complication being the need for a postoperative blood transfusion. Consequently, current standard practice involves cross-matching two units of red blood cells for each patient preoperatively. This incurs significant costs and is associated with blood product complications for transfused patients who are at low risk. This study aims to identify factors that could increase the risk of requiring a blood transfusion, thereby facilitating the adaptation of preoperative cross-matching practices to reflect individual patient risks rather than using a generalised approach.

Methods: A retrospective analysis was conducted of 319 patients from a major acute teaching hospital between June 2023 and January 2024, examining risk factors including preoperative haemoglobin levels, age, gender, type and duration of surgery, type of fracture, BMI, use of anticoagulants, and comorbidities (diabetes, hypertension, cancer, chronic obstructive pulmonary disease (COPD)/asthma, heart disease). Binary logistic regression and odds ratios were used to assess their impact on blood loss and transfusion need.

Results: Ninety-nine patients (31%) in our cohort received a blood transfusion. Preoperative haemoglobin (p<0.001) and age (p=0.039) were the only factors found to be significant predictors of the likelihood of needing a blood transfusion. The exponentiation of the B coefficient (Exp(B)) values were 0.920 and 1.040, respectively, indicating a negative correlation for preoperative haemoglobin and a positive correlation for age. Statistical analysis between the group receiving a transfusion versus those who did not showed that patients were significantly older (p<0.001), had lower BMI (p=0.037), and had lower preoperative haemoglobin (p<0.001) in the transfused group. Odds ratios indicated that females (1.34) and patients with hypertension (1.19), cancer (1.09), COPD or asthma (1.06), and heart disease (1.45) were more likely to receive transfusions. Intracapsular fractures (undisplaced 0.12, displaced 0.38) and arthroplasty (0.38) showed lower odds compared to intramedullary (IM) nailing (3.13). The odds ratio changes to less than one for haemoglobin values >110 g/L (<0.7) and increases to values greater than one for age above 80 (>1.27).

Conclusion: Increased age and low preoperative haemoglobin significantly raise the risk of hidden blood loss and transfusion need following neck of femur surgery. We suggest further development of a risk prediction model to improve surgical outcomes, reduce healthcare costs, and optimise resource utilisation.

## Introduction

Hip fractures are a frequent cause of surgical admission among the elderly [1], with approximately 76,000 cases annually in the UK requiring surgical intervention [2]. A notable complication of hip fracture surgery is the need for a blood transfusion. As a result, informed consent for transfusions is routinely obtained from patients undergoing the consent process for neck of femur surgeries, should the need arise for a transfusion after surgery. To prepare for this risk, a group and save blood test is conducted preoperatively to determine the patient’s blood type and the presence of atypical antibodies [3]. The plasma and red blood cells are then separated and stored to facilitate re-examination in the event of a transfusion-related complication [4].

Although no specific national guidelines mandate a group and save test for every patient, local guidelines, such as those from South Tees National Health Service (NHS) Foundation Trust, recommend it as part of the preoperative protocol for hip fracture patients [5]. Furthermore, the Maximum Surgical Blood Ordering Schedule (MSBOS) advises that patients undergoing neck of femur and femoral shaft fracture surgeries should have a group and save test and two units of red blood cells cross-matched [6].

In the 2017 annual report from the National Hip Fracture Database, it was estimated that hip fractures cost the NHS up to £1 billion annually [7]. According to a study by Quinn et al., the cost of a group and save blood test is £20 [8]. Given the assumption that there are 76,000 hip fractures each year and that each patient undergoes a group and save on presentation, the total annual cost for this blood test is £1,520,000. The cost of one unit of red blood cells (RBC) is estimated at £240.90 [9]. If each patient is cross-matched for two units preoperatively, the total cost for blood products amounts to £36,616,800 annually. However, not all patients receive the cross-matched blood, as their haemoglobin levels may not fall to the threshold requiring transfusion. National Institute for Health and Care Excellence (NICE) guidelines on blood transfusion, as used in our institution, state that a threshold of 70 g/L should be used when assessing the need for transfusion [10]. As a result, unused cross-matched blood must be recycled for reallocation to other patients. This reallocation process incurs additional costs, thereby increasing the overall expenses of the cross-matching process. Considering all these factors, the total costs for group and save and cross-matching blood amount to £38,136,800 annually. This represents nearly 4% of the total costs associated with hip fractures.

Blood transfusions additionally entail significant risks, including reactions, infections, and haemolysis [11]. They have been associated with a 1.9-fold increase in morbidity [12]. In various surgical contexts, time-delayed postoperative transfusions correlate with higher rates of readmission and postoperative complications [13].

Is it necessary to perform blood tests and cross-matching for all patients, or can this process be risk-stratified? By implementing risk stratification, resources such as time and money could be allocated more efficiently by focusing on patients with a higher risk of requiring a transfusion and reducing unnecessary procedures for those with a lower risk.

This review examines risk factors in order to develop a risk prediction score for transfusion need in neck of femur surgeries. This can streamline the cross-matching process, reduce unnecessary costs and transfusions, minimise associated complications, and improve patient outcomes by enabling early identification and close monitoring of those at high risk of requiring a transfusion.

## Materials and methods

Cohort selection and data collection

This study utilised retrospective data from a major acute teaching hospital to form a cohort of patients with neck of femur fractures between June 2023 and January 2024. The data for these patients were initially found in the National Hip Fracture Database, with additional data subsequently obtained from patient records. Multiple risk factors were analysed for their impact on hidden blood loss and the need for transfusion, including preoperative haemoglobin level, age, gender, fracture type, surgery type and duration, BMI, anticoagulant use, and comorbidities such as diabetes, hypertension, cancer, chronic obstructive pulmonary disease (COPD) or asthma, and heart disease.

Associations between certain risk factors and blood loss have been evidenced in previous studies. Preoperative haemoglobin was associated with blood loss in major femoral head and neck surgery [14], while BMI and surgical duration were associated with increased blood loss in lumbar fusion surgery [15]. Age was also carefully examined, as it has an established correlation with the risk of acquiring a hip fracture [16]. Anticoagulant use was analysed in the context of hip fractures due to its association with adverse outcomes in bariatric surgery in another study [17]. Lastly, comorbidities common in elderly patients were investigated due to the prevalence of hip fractures in this demographic [18].

Exclusions from the cohort included patients undergoing total hip replacement, those with periprosthetic or femoral shaft fractures, and those with multiple concurrent surgeries. This was done to avoid skewed results from the anticipated higher transfusion need.

Analysis of data

The collected data were compiled into an anonymised spreadsheet and imported into IBM SPSS Statistics for Windows, Version 29 (Released 2023; IBM Corp., Armonk, New York) for analysis.

Descriptive statistics were gathered using SPSS to compare data between two groups: those who received a blood transfusion and those who did not. Continuous variables were analysed for central tendency and variance, with normality assessed via the Shapiro-Wilk test and histogram visualisation. The mean was recorded for continuous data with normal distributions, while the median was recorded for continuous data with non-normal distributions. Categorical variables were summarised by frequencies and percentages.

Statistical comparisons between the two groups were conducted using independent t-tests for normally distributed continuous data, Mann-Whitney U tests for non-normally distributed continuous data, and Fisher’s exact or Pearson’s chi-squared tests for dichotomous and non-dichotomous categorical data, respectively. A 5% significance level was applied, with p-values <0.05 indicating statistical significance.

For the primary analysis, binary logistic regression was employed to identify variables significantly influencing the likelihood of receiving a blood transfusion. The dependent variable was transfusion status, while the independent variables included the risk factors previously mentioned. For the multiple-category variables "type of fracture" and "type of surgery," "intracapsular-displaced" and "arthroplasty" were selected as the reference categories, respectively. "Male" served as the reference category for gender, allowing for comparison to females. A 5% significance level was also applied. The regression output included the exponential of the variable (Exp(B)), indicating the effect size with a 95% confidence interval (CI).

An odds ratio was calculated for both continuous and categorical variables to illustrate their directional impact. Continuous data were binned into categories. Preoperative haemoglobin and age were grouped into decadal ranges, while BMI was classified as underweight, healthy weight, and obese.

Finally, collinearity was examined using SPSS to assess the relationship between the statistically significant variables. This analysis was conducted by performing a binomial Pearson correlation test.

## Results

Descriptive statistics

The final cohort comprised 319 patients, with 99 (31%) receiving blood transfusions and 220 not receiving transfusions.

Table 1 presents the continuous data with means and their standard deviations in parentheses for normal distributions, and medians with their interquartile ranges (25th and 75th percentiles) in parentheses for non-normal distributions. An independent t-test was conducted to compare the mean ages of the two groups, yielding a p-value of 0.001, indicating that patients in the transfused group were significantly older than those in the non-transfused group. The following p-values were demonstrated for the non-normally distributed continuous variables: p<0.001 for preoperative haemoglobin, demonstrating a significant difference with lower haemoglobin levels in the transfused group; p=0.037 for BMI, indicating a statistically significant difference with the transfused group having lower BMI; and p=0.618 for the duration of surgery, which showed no significant difference between the two groups.

**Table 1:** Central tendency and variance of continuous variables: transfused vs not transfused Means and their standard deviations in parentheses for normal distributions. Medians with their interquartile ranges (25th and 75th percentiles) in parenthesis for non-normal distributions.

Variable	Transfused, n=99	Not transfused, n=220	t-test or Mann-Whitney U test	Significance
Pre-operative haemoglobin (g/L)	108.00 (99.00, 123.00)	128.91 (119.00, 138.00)	4468.5	<0.001
Age	83.66 (8.49)	80.21 (8.97)	-3.223	0.001
BMI	21.98 (19.23, 25.13)	23.08 (20.24, 26.32)	9298.0	0.037
Duration of surgery	2:28 (2:03, 2:59)	2:29 (2:02, 2:50)	10510.5	0.618

Table 2 displays categorical data with frequencies and their percentages in parentheses. Statistical analysis revealed significant differences between the groups for both type of fracture and type of surgery (p<0.001). The remaining variables showed no significant differences, with p-values greater than 0.05, as indicated in the final column of Table 2.

**Table 2:** Frequencies and percentages of categorical variables: transfused vs not transfused Frequencies and their percentages in parentheses. IM: intramedullary, COPD: chronic obstructive pulmonary disease.

Variable		Transfused, n=99	Not transfused, n=220	Fisher Exact or Pearson chi-squared coefficient	Significance
Gender	Male	28 (28.3)	76 (34.5)	1.219	0.303
	Female	71 (71.7)	144 (65.5)		
Type of fracture	Intracapsular – undisplaced	1 (1.0)	17 (7.7)	25.877	<0.001
	Intracapsular – displaced	32 (32.3)	121 (55.0)		
	Trochanteric – grade A1/A2	43 (43.4)	57 (25.9)		
	Trochanteric – grade A3	16 (16.2)	17 (7.7)		
	Subtrochanteric	7 (7.1)	8 (3.6)		
Type of surgery	Arthroplasty	33 (33.3)	119 (54.1)	18.240	<0.001
	Fixation	32 (32.3)	66 (30.0)		
	IM nail	34 (34.3)	35 (15.9)		
Diabetes	Diabetic	16 (16.2)	41 (18.6)	0.285	0.639
	Not diabetic	83 (83.8)	179 (81.4)		
Hypertension	Hypertensive	51 (51.5)	106 (48.2)	0.304	0.629
	Not hypertensive	48 (48.5)	114 (51.8)		
Cancer	Cancer	26 (26.3)	54 (24.5)	0.107	0.781
	No cancer	73 (73.7)	166 (75.5)		
COPD/asthma	COPD or asthma	24 (24.2)	51 (23.2)	0.043	0.887
	No COPD or asthma	75 (75.8)	169 (76.8)		
Heart disease	Heart disease	29 (29.3)	48 (21.8)	2.083	0.159
	No heart disease	70 (70.7)	172 (78.2)		
Use of anticoagulants	Using anticoagulants	22 (22.2)	44 (20.0)	0.205	0.377
	Not using anticoagulants	77 (77.8)	176 (80.0)		

Binary logistic regression

A binary logistic regression of each variable individually on the likelihood of receiving a transfusion is presented in Table 3. The results indicate that preoperative haemoglobin and age are statistically significant predictors of the likelihood of receiving a transfusion, with p-values of p<0.001 and p=0.039, respectively. The direction and magnitude of these effects are reflected in the Exp(B) values, which are 0.920 for preoperative haemoglobin, indicating a negative relationship, and 1.040 for age, indicating a positive relationship. None of the other variables demonstrated individual statistical significance.

**Table 3:** Binary logistic regression of all variables showing the significance of their impact on receiving a blood transfusion Exp(B): exponentiation of the B coefficient, CI: confidence interval, IM: intramedullary, BMI: body mass index, COPD: chronic obstructive pulmonary disease.

Variable	Significance	Exp(B)	Lower CI	Upper CI
Preoperative haemoglobin (g/L)	<0.001	0.920	0.899	0.942
Age	0.039	1.040	1.002	1.079
Gender	0.911	0.962	.489	1.893
Type of fracture	0.367	-	-	-
Intracapsular – undisplaced	0.252	0.293	0.021	2.765
Trochanteric A1/A2	0.319	3.466	0.301	39.972
Trochanteric A3	0.359	3.353	0.253	44.459
Subtrochanteric	0.356	3.754	0.226	62.406
Type of surgery	0.511	-	-	-
Fixation	0.841	0.781	0.07	0.104
IM Nail	0.825	1.333	0.104	17.125
Duration of surgery	0.130	1.000	1.000	1.000
BMI	0.641	0.984	0.919	1.053
Diabetes	0.112	0.506	0.219	1.173
Hypertension	0.835	0.936	0.500	1.751
Cancer	0.906	1.042	0.527	2.063
COPD/asthma	0.468	1.314	0.628	2.749
Heart disease	0.243	1.559	0.740	3.288
Use of anticoagulants	0.833	0.921	0.430	1.976

Odds ratios

Odds ratios are presented in Table 4. The odds ratio compares the likelihood of individuals in specific categories or bins of continuous data of a variable receiving a transfusion versus not receiving a transfusion. An odds ratio value of 1 indicates no difference in odds between the two groups; a value greater than 1 suggests the event is more likely in the transfused group compared to the non-transfused group; and a value less than 1 indicates the event is more likely in the non-transfused group than in the transfused group. For binary categories, females exhibit an odds ratio greater than 1 (1.34), whereas males have an odds ratio less than 1 (0.75). Conditions such as hypertension (1.19), cancer (1.09), COPD or asthma (1.06), heart disease (1.45), and anticoagulant use (1.14) also have odds ratios greater than 1. Conversely, diabetes shows an odds ratio of 0.84, and the absence of diabetes has an odds ratio of 1.19. Intracapsular fractures have odds ratios less than 1 (0.12 and 0.38 for undisplaced and displaced fractures, respectively), while other fracture types have odds ratios greater than 1. The odds ratio for IM nailing and fixation exceeds 1 (3.13 and 1.09, respectively), whereas arthroplasty is associated with an odds ratio of 0.38.

Preoperative haemoglobin levels below 110 g/L have odds ratios greater than 1 (3.97 for 101-110 g/L, 66.28 for 91-100 g/L, and infinity for 81-90 g/L), whereas higher levels show odds ratios less than 1 (0.7 for 111-120 g/L, 0.15 for 131-140 g/L, and onwards in Table 4). Age categories up to 80 years have odds ratios less than 1 (0.39 for <70, 0.69 for 71-80), while those above 80 show odds ratios greater than 1 (1.27 for 81-90, 2.08 for >91). Regarding BMI, underweight and healthy weight categories have odds ratios greater than 1 (1.31 and 1.03, respectively), whereas obesity has an odds ratio of 0.31. Lastly, the duration of surgery does not exhibit a consistent trend, with odds ratios fluctuating above and below 1 across its different categories.

**Table 4:** Odds ratios and CI of each category or bin of all variables CI: confidence interval, IM: intramedullary, BMI: body mass index, COPD: chronic obstructive pulmonary disease.

Variable		Odds ratio	Lower CI	Upper CI
Preoperative haemoglobin (g/L)	81–90	Infinity	Infinity	Infinity
	91–100	66.28	8.8	499.14
	101–110	3.97	2.08	7.57
	111–120	0.7	0.36	1.35
	121–130	0.65	0.36	1.2
	131–140	0.15	0.06	0.38
	141–150	0.5	0.16	1.53
	151–160	0	0	0
	161–170	0	0	0
	171–180	0	0	0
Age	<70	0.39	0.17	0.91
	71–80	0.69	0.41	1.17
	81–90	1.27	0.78	2.05
	>91	2.08	1.13	3.83
Gender	Female	1.34	0.8	2.25
	Male	0.75	0.45	1.25
Type of fracture	Intracapsular – undisplaced	0.12	0.02	0.93
	Intracapsular – displaced	0.38	0.23	0.63
	Trochanteric A1/A2	2.2	1.33	3.62
	Trochanteric A3	2.3	1.11	4.77
	Subtrochanteric	2.21	0.78	6.26
Type of surgery	Arthroplasty	0.38	0.23	0.63
	Fixation	1.09	0.65	1.82
	IM nail	3.13	1.81	5.41
Duration of surgery	<1:30	0.73	0.23	2.32
	1:31–2:00	0.81	0.42	1.59
	2:01–2:30	1.49	0.9	2.44
	2:31–3:00	0.66	0.37	1.15
	3:01–3:30	1.22	0.63	2.36
	>3:31	1.12	0.41	3.07
BMI	<24.99	1.31	0.77	2.23
	25–29.99	1.03	0.59	1.81
	>30	0.31	0.09	1.08
Diabetes	Diabetic	0.84	0.45	1.59
	Not diabetic	1.19	0.63	2.24
Hypertension	Hypertensive	1.19	0.74	1.91
	Not hypertensive	0.84	0.52	1.36
Cancer	Cancer	1.09	0.64	1.88
	No cancer	0.91	0.53	1.57
COPD/asthma	COPD/asthma	1.06	0.61	1.85
	No COPD/asthma	0.94	0.54	1.64
Heart disease	Heart disease	1.45	0.85	2.47
	No heart disease	0.69	0.4	1.18
Use of anticoagulants	Using anticoagulants	1.14	0.64	2.04
	Not using anticoagulants	0.88	0.49	1.56

Correlation

As indicated, preoperative haemoglobin (p<0.001) and age (p=0.039) were the two significant variables. To ensure the stability of the regression model, it was necessary to assess collinearity to determine if these variables were correlated. This yielded a correlation coefficient of -0.106 with a p-value of p=0.004.

## Discussion

The aim of this research was to identify factors contributing to blood transfusions following neck of femur fracture surgeries. The results indicated that both preoperative haemoglobin levels and patient age significantly influenced this outcome. While none of the other variables showed individual statistical significance, their directional impact was demonstrated through odds ratios.

Binary logistic regression

For preoperative haemoglobin, the Exp(B) value demonstrates that an increase of one unit in preoperative haemoglobin corresponds to a decrease in the odds of requiring a transfusion by a factor of 0.920. Thus, lower preoperative haemoglobin levels are associated with a higher risk of transfusion.

Conversely, the Exp(B) value for age demonstrates that for each additional year of age, the odds of receiving a transfusion increase by a factor of 1.040, raising the risk as age advances. The remaining variables had p-values exceeding 0.05, indicating they did not significantly influence the likelihood of receiving a blood transfusion.

The above analyses were univariate in nature and did not examine interactions between variables. It is plausible that these variables do not act in isolation; for example, the risk associated with low preoperative haemoglobin may be compounded by the presence of comorbid conditions such as hypertension. In future research, incorporating interaction terms within the analytical model may be warranted to elucidate the combined effects of multiple variables on the outcomes.

Odds ratio 

Haemoglobin levels below 110 g/L are associated with a higher likelihood of requiring a transfusion, whereas levels above 111 g/L are associated with a lower likelihood. For age, patients older than 80 years are more likely to require a transfusion, while those younger than 80 are less likely.

For variables that were not statistically significant, caution is warranted when interpreting the odds ratios, though they can still indicate directional impacts. For instance, the results for BMI indicate an increased risk of transfusion for individuals who are underweight or of healthy weight, whereas obese individuals were less likely to require a transfusion. The duration of surgery did not show a consistent trend related to the risk of transfusion. In the categorical data, patients with hypertension, cancer, COPD or asthma, heart disease, or those using anticoagulants exhibited a higher likelihood of needing a transfusion. Conversely, diabetes appeared to have a protective effect, as diabetic patients were less likely to require a transfusion.

In fracture types, intracapsular fractures presented a significantly lower risk of transfusion compared to trochanteric and subtrochanteric fractures, with the trochanteric A3 type exhibiting the highest risk. The IM nail was associated with the highest risk in surgical procedures, followed by fixation, while arthroplasty was linked to a lower risk of requiring a transfusion.

Correlation 

The negative correlation coefficient (-0.106) between the two significant variables, preoperative haemoglobin and age, indicates a slight inverse relationship between them, such that as one variable increases, the other tends to decrease. However, this correlation coefficient is close to zero, suggesting a weak relationship between the variables and is of limited practical significance. Consequently, the absence of substantial collinearity between the variables indicates that they can be analysed independently of one another, and both variables contribute significantly to the outcome in their own right.

Implications 

As previously noted, routine blood tests and cross-matching are standard practices for all patients undergoing this procedure. We aimed to evaluate whether these practices are necessary for every patient or if a risk-stratified approach could be more efficient.

Knowing that age is a significant risk factor that increases the likelihood of transfusion, as indicated in this study, highlights the importance of enhanced monitoring of haemoglobin levels and potential blood loss in older patients following surgery. Such proactive monitoring could facilitate earlier transfusions and potentially improve patient outcomes.

For patients presenting with preoperative anaemia, addressing and correcting low haemoglobin levels prior to surgery could mitigate the risk and subsequent need for postoperative transfusions. Consistent with prior research that identified preoperative haemoglobin as a risk factor for blood transfusions after major head and neck surgeries [14], this study corroborates these findings in the context of neck of femur repairs.

This research lays the groundwork for further investigations and the potential development of a predictive model for assessing the risk of transfusion following neck of femur repairs. Such a model could assign risk scores based on various risk factors. For instance, a preoperative haemoglobin level below 90 g/L might receive a score of three points, levels between 90-100 g/L could receive two points, and levels from 101-110 g/L could receive one point, with additional points allocated for other risk factors. This scoring system would allow for a risk assessment that predicts the likelihood of requiring a blood transfusion. Should the model demonstrate that patients with low or zero risk scores do not require group and save blood tests or cross-matched RBC units, it could lead to substantial cost savings for the National Health Service (NHS). This would underscore the potential benefits of adopting a more targeted risk-based approach to preoperative blood management in hip fracture patients.

Strengths**

A notable strength of this study is the size and composition of the patient cohort. The study included 319 patients, 99 of whom received blood transfusions, providing a sufficiently large sample to enhance the generalisability of the results. Moreover, the cohort comprised both male and female patients across a broad age range, thereby making the findings more representative of the general population.

Limitations**

As an observational study, a limitation of this analysis is its selection of multiple variables without prior hypotheses regarding their impact or significance. To address this, future research should focus on the significant variables identified and seek to mitigate their effects to reduce the risk of transfusion in neck of femur repair surgeries.

Another limitation pertains to the cohort composition, as ethnicity data were not recorded. This omission restricts our ability to fully assess the representativeness of the cohort concerning ethnic diversity, potentially affecting the generalisability of the findings. Additionally, blood loss due to surgery itself was not precisely quantified in our study in relation to transfusion requirements, limiting our capacity to directly correlate measured blood loss with the need for transfusion.

Despite these limitations, the study provides valuable insight into the identification of risk factors for blood transfusion need, with two variables demonstrated to have significant independent effects. The research underscores the need for further investigation to expand our understanding of these factors to improve patient outcomes while also potentially reducing costs for the NHS.

## Conclusions

In conclusion, this study identified that increased age and low preoperative haemoglobin are associated with a higher risk of requiring a blood transfusion following neck of femur fracture surgery. Further research is warranted to examine these variables in conjunction, with the objective of developing a risk prediction model to enhance surgical outcomes for future patients undergoing neck of femur repairs. Such a model also has the potential to reduce healthcare costs and enable more effective resource allocation.
